# Drug-induced resistance evolution necessitates less aggressive treatment

**DOI:** 10.1371/journal.pcbi.1009418

**Published:** 2021-09-23

**Authors:** Teemu Kuosmanen, Johannes Cairns, Robert Noble, Niko Beerenwinkel, Tommi Mononen, Ville Mustonen

**Affiliations:** 1 Organismal and Evolutionary Biology Research Programme, Department of Computer Science, University of Helsinki, Helsinki, Finland; 2 Department of Biosystems Science and Engineering, ETH Zurich, Basel, Switzerland; 3 Department of Evolutionary Biology and Environmental Studies, University of Zurich, Zurich, Switzerland; 4 Present address: Department of Mathematics, City, University of London, London, United Kingdom; 5 Institute of Biotechnology, Helsinki Institute for Information Technology, University of Helsinki, Helsinki, Finland; University of New South Wales, AUSTRALIA

## Abstract

Increasing body of experimental evidence suggests that anticancer and antimicrobial therapies may themselves promote the acquisition of drug resistance by increasing mutability. The successful control of evolving populations requires that such biological costs of control are identified, quantified and included to the evolutionarily informed treatment protocol. Here we identify, characterise and exploit a trade-off between decreasing the target population size and generating a surplus of treatment-induced rescue mutations. We show that the probability of cure is maximized at an intermediate dosage, below the drug concentration yielding maximal population decay, suggesting that treatment outcomes may in some cases be substantially improved by less aggressive treatment strategies. We also provide a general analytical relationship that implicitly links growth rate, pharmacodynamics and dose-dependent mutation rate to an optimal control law. Our results highlight the important, but often neglected, role of fundamental eco-evolutionary costs of control. These costs can often lead to situations, where decreasing the cumulative drug dosage may be preferable even when the objective of the treatment is elimination, and not containment. Taken together, our results thus add to the ongoing criticism of the standard practice of administering aggressive, high-dose therapies and motivate further experimental and clinical investigation of the mutagenicity and other hidden collateral costs of therapies.

## Author summary

Evolution of drug resistance to anticancer and antimicrobial therapies is widespread among cancer and pathogen cell populations. Classical theory posits strictly that genetic and phenotypic variation is generated in evolving populations independently of the selection pressure. However, recent experimental findings among antimicrobial agents, traditional cytotoxic chemotherapies and targeted cancer therapies suggest that treatment not only imposes selection but can also affect the rate of adaptation by increasing mutability. Here we analyse a model with drug-induced increase in mutation rate and explore its consequences for treatment optimisation. We argue that the true biological cost of treatment is not limited to the harmful side-effects, but instead realises even more profoundly by fundamentally changing the underlying eco-evolutionary dynamics within the microenvironment. Using the concept of evolutionary rescue, we formulate the treatment as an optimal control problem and solve the optimal elimination strategy, which minimises the probability of evolutionary rescue. We show that aggressive elimination strategies, which aim at eradication as fast as possible and which represent the current standard of care, can be detrimental even with modest drug-induced increases (fold change ≤10) to the baseline mutation rate.

## Introduction

The formation of cancer and emergence of antimicrobial resistance (AMR) are notorious examples of fast paced evolution. Modern medicine has developed various drugs to target cancer and pathogen cell populations with the aim to drive them to extinction using aggressive, high-dose therapies. However, these treatments frequently fail due to drug resistance, a phenomenon where the drug loses its desired pharmacodynamical effects. The emergence of drug resistance is the consequence of evolution which continues also during the treatment. Indeed, the administration of treatment represents a major switch point in the evolutionary trajectories of these populations, initiating a rapid phase of human-induced evolution.

The most desirable consequence of this treatment dynamics is the decay of the drug-sensitive target population. The key question in such a situation is whether adaptive evolution can happen fast enough to save the population from extinction. If the population is saved, we say that an *evolutionary rescue* has occurred [1]. Introduced first in the field of conservation biology, where the objective has been to design the most efficient intervention programs to save endangered species from going extinct, the concept of evolutionary rescue can be readily applied also to the study of drug resistance [2, 3] with the opposite goal in mind.

Evolutionary rescue can occur either by standing variation or by *de novo* mutation. Rescue by standing variation corresponds to the intrinsic resistance model in which the population is sufficiently diverse to contain individuals that can survive in the changed environment. Rescue by *de novo* mutation, on the other hand, corresponds to the acquired resistance model in which (partially-)resistant individuals are created by mutational processes after the initiation of therapy. The deeply rooted paradigm of administering treatment as aggressively as possible to maximize cell kill [4] has its origin in the somatic mutation theory of drug resistance [5], where it is assumed that rescue mutations arise spontaneously and independently of the treatment. The rationale of such aggressive elimination therapies is then to maximize the probability of cure by eradicating the population as fast as possible thus minimising the rescue window during which mutations can occur and save the population.

However, the gain of population decay comes necessarily with a cost, which realizes as collateral damage at various scales. The most obvious examples of such damage are the clinical side-effects of the treatment, which often result from the off-target exposure to the drug. For example, traditional anticancer therapies hit also healthy tissues while antimicrobial agents negatively affect the natural gut microbiome. The detrimental side-effects experienced by the patient yield a toxicity constraint which have led to the maximum tolerated dose (MTD) paradigm [6], where treatment is predominantly administered at the highest cumulative dose possible given the toxicity constraint.

Recent research and experimental evidence suggest that the true biological cost of the treatment is not limited to the harmful side-effects, but instead realize even more profoundly by fundamentally altering the underlying eco-evolutionary dynamics within the microenvironment. The harsh selection pressure induced by the treatment not only leads to the decay of the sensitive cells, but it can also enhance the growth opportunities of the pre-existing or emerged resistant cells, a well-known phenomenon of competitive release [4, 7]. In these cases, aggressive chemotherapy can accelerate the population’s evolution towards treatment resistance as by removing the competing sensitive cells the resistant cells have even more resources to reoccupy the niche leading to relapse.

This problem has then motivated various authors to suggest so-called containment strategies which use the minimal amount of control to keep the population burden in check while deliberately maintaining sensitive cells to competitively suppress the growth of the existing resistant cells as a form of ecological control [8–11]. Competitive release represents an important ‘ecological collateral damage’ of treatment, which promotes the emergence of drug resistance and leads to treatment failure. Besides the altered competition dynamics, beneficial rescue mutations may become enriched in the off-target species and promote the emergence of AMR by means such as horizontal gene-transfer [12].

In addition to the extensive ecological consequences, the treatment may also induce changes to the intrinsic dynamics of the target cells other than Darwinian selection. Such ‘evolutionary collateral damage’ can realize, for example, by the treatment enhancing the evolvability of the population. Classical theory, based on the famous Luria-Delbrück experiments [13] and somatic mutation theory of drug resistance, posits strictly that genetic and phenotypic variation is generated independently of the selection pressure [14]. In contrast, recent experimental evidence suggests that the roles of mutation and selection cannot be fully separated from each other as the drug therapies themselves may affect the way variation is generated. Studies in bacteria demonstrate that stress alone can increase the genome-wide mutation rate, driven by switch to more error-prone DNA repair mechanisms [15]. Recently, similar findings were reported also in cancer in the context of targeted cytostatic therapies [16, 17]. Higher levels of reactive oxygen species during the treatment may serve as another mechanism which increases the mutation rate [18]. Besides the direct stress-induced mutation-rate plasticity, the spontaneous mutation rate could be modulated also indirectly via the population density, where lower population densities seem to be strongly correlated with higher mutation rates [19]. On the other hand, conventional genotoxic chemotherapies may also cause specific drug-induced mutations, such as the reported distinct mutational signatures of platinum-based therapies [20].

Secondly, natural selection can give rise to resistant phenotypes even in the absence of genetic mutations as certain resistance mechanisms can be activated using epigenetic regulation as a form of Lamarckian induction [21]. Cancer cells—especially stem-like cancer cells—can exhibit diverse phenotypic plasticity, dynamically responding to changes in their environment. Cells can enhance their survival via life-history trade-offs by reallocating resources normally devoted to proliferation [22, 23]. Such quiescent, drug-tolerant cells can act as a reservoir from which permanently resistant cells can emerge via further genetic mutations, or alternatively, revert back to active proliferation upon treatment discontinuation [24]. Similar phenomena are also widespread in bacterial populations whereby a subset of the population expresses resistance-conferring efflux pumps as a function of drug exposure and concentration, in turn, allowing survival and reproduction until the occurrence of resistance mutations [25–27]. Drug-induced phenotypic switching may thus significantly contribute to evolutionary rescue in a dose-dependent fashion either by buying more time for adaptive evolution or even by being a self-sufficient adaptation itself.

Previous and other drug exposures may also influence the bacterial physiological response and resistance trajectory to a particular drug. Resistance against one antibiotic may be associated with, long or short term, collateral sensitivity or resistance to other drugs showing another form of therapy induced collateral damage and an opportunity for therapy optimisation. For instance, collateral sensitivity profiles can be used to address the trade-off between maintaining long-term sensitivity of the target population at the expense of short-term periods of high resistance [28]. Or cellular hysteresis, which is the transgenerational change in cellular physiology that is induced by one antibiotic and sensitizes bacteria to another subsequently utilized antibiotic can be exploited by using fast sequential treatments [29].

Finally, the phenotypic mutation rate can also change due to the dose-dependency of the required mutational targets [30]. Higher levels of stress can decrease the phenotypic mutation rate as the target size for a single sufficient mutation could decrease, and because multiple mutations may be required for the resistant phenotype. Alternatively, the target size may also increase within some concentration ranges if the proportion of beneficial mutations increases as the selection pressure becomes harsher. This way drug resistance may still emerge by gradual stepwise adaptations, especially in drug sanctuaries [31]. Thus, even if the required evolutionary distance increases with the drug concentration, this could be more than offset by the higher mutational supply, stronger selection and resistance induction. All these findings provide compelling evidence that the rate of adaptation to the stressful environment is strongly dose-dependent and not constant.

The outlined collateral damage occurring at various scales ranging from the whole patient to the microenvironment of the target cells greatly complicate the combat against drug resistance and require the integration of ecological and evolutionary dynamics into therapy design. Eco-evolutionary control has to further factor in the underlying biological mechanism of control [32]. Therapy is often based on biomolecular interactions, such as drug–target or antibody–antigen binding [33]. The biophysics of molecular binding dictate a finite control leverage, for an example, as seen in the Hill-function type pharmacodynamics which cause a saturation of the drug’s effect. However, the different collateral damage caused by the drug do not saturate in general, but can keep increasing with the dosage, or saturate at a different concentration. These observations point to a great scope in designing treatments using eco-evolutionary control theory [32].

Majority of previous treatment optimisation models have focused on optimising the delivery mode with respect to some toxicity constraint (see e.g. [34]). The “second-wave” of treatment optimisation has focused on the issue of competitive release and the investigation of various containment strategies [11, 35]. Here we investigate the consequences of evolutionary collateral damage, as realized by drug-induced resistance evolution, on treatment selection using the rigorous methods of optimal control theory [36].

Only few previous theoretical studies of drug resistance have explicitly accounted for dose-dependent mutation rates [37–39]. Liu *et al.* [37] find that the optimal delivery mode (e.g. pulsed or continuous) of the MTD-strategy is robust against changes in the mutation rate in a model for targeted cytostatic cancer therapy. Greene *et al.* [38] on the other hand show that the dose-dependent mutation rate may have a significant impact on which delivery mode is preferable (for more rigorous mathematical treatment of the presented model see [39]). In contrast, we solve the optimal elimination strategy, which minimises the probability of evolutionary rescue, and demonstrate the potential to improve treatment outcomes by reducing the total cumulative dosage administered.

We identify and exploit a trade-off, where increasing the dosage reduces *de novo* mutations by decreasing the target population size faster, but at the expense of simultaneously generating a surplus of treatment-induced mutations. By simulating virtual treatments *in silico*, we show that the probability of cure changes non-monotonically as a function of the drug concentration and is maximized at an intermediate dosage. Our results highlight the importance of dose-dependencies in resistance evolution and help to redefine the precise evolutionary objectives of the treatment, providing a framework for systematic therapy optimisation.

## Results

### Dose-dependent mutation rate introduces a trade-off between mutation intensity and target population decay

Here we set the therapy optimisation problem by formulating the specific control objectives that we want to achieve while factoring in the eco-evolutionary dynamics of the target population. We further demonstrate how drug-induced mutations affect realising these goals.

**Fig 1:**
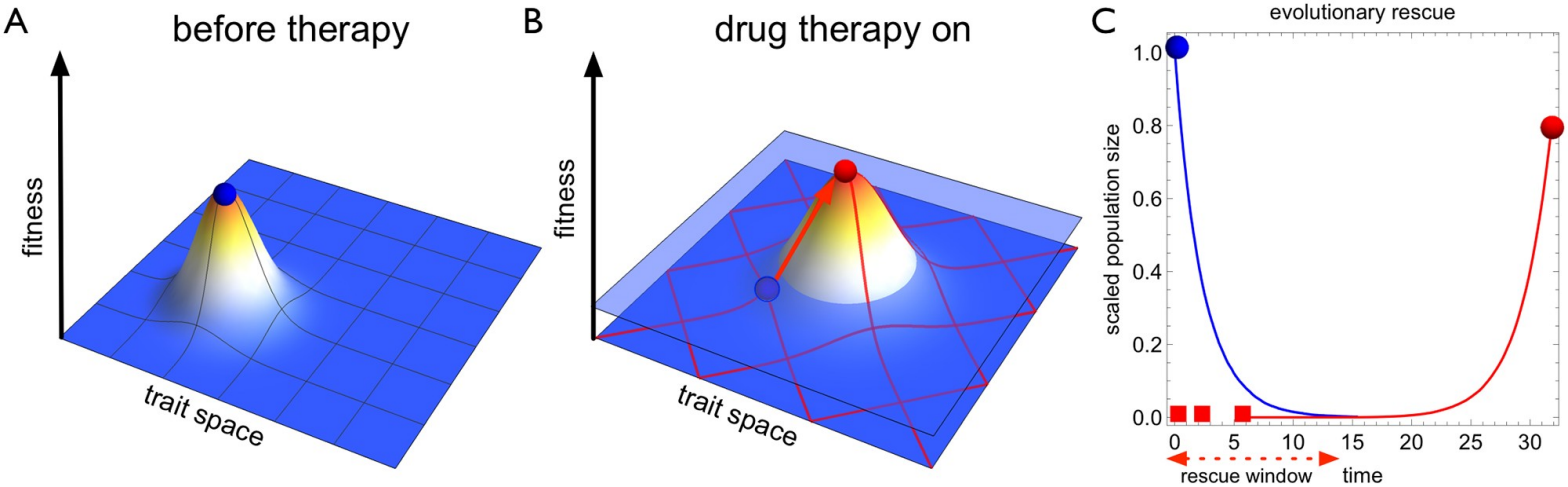
Drug-induced mutations realise an evolutionary collateral cost of therapy. **A** Before therapy the target cell population (blue circle) is well-adapted to its microenvironment. **B** Initiation of control (drug therapy) drastically changes the growth conditions of the target population pushing it below zero level of growth (light blue plane). At the same time opportunities to adapt to the new conditions create a selection pressure for resistance to evolve. Furthermore, the therapy can change the mutational wiring both qualitatively and quantitatively (red mesh). The effect of therapy on the mutational processes represent an evolutionary collateral damage of control, which can expedite the emergence of resistance (red arrow and circle). **C** The treatment eliminates the sensitive cell population (blue) but an evolutionary rescue can occur if a resistant mutant (red squares) manages to successfully establish during the rescue window. Here we derive optimal treatment strategies that minimise the probability of evolutionary rescue while taking into account drug-induced mutations.

Fig 1C provides a schematic example of an evolutionary rescue: first, the population rapidly declines as the treatment eradicates sensitive cells. Cells can however acquire mutations that reduce their sensitivity to the drug. The emergent resistant cells are strongly favoured by natural selection but can nevertheless be lost due to stochastic extinction [40]. If resistant cells manage to establish, they will soon repopulate the released niche and rescue the population from going extinct.

We use the term *rescue window* for the initial treatment period during which sensitive cells can acquire mutations and the population can be rescued. We model the acquisition of rescue mutants by a time-inhomogeneous Poisson process during the rescue window, where the rate of gaining a new mutant at time *t* is given by the product of the (sensitive) cell population size *S*(*t*, *u*(*t*)) and the phenotypic mutation rate *μ*(*u*(*t*)). Both of these factors depend on the drug concentration *u*(*t*), as we explicitly take into account drug-induced effects. Because the growth of resistant cells originates from a single cell, we must use stochastic population dynamics to model the growth of small populations that have a considerable extinction risk due to inherent stochastic fluctuations. The stochastic extinction risk for a simple birth-death process founded by a single resistant (subscript *R*) cell is given by $q=\frac{\delta_{R}}{\beta_{R}}$ (see e.g. [41]), where *δ*_*R*_ and *β*_*R*_ are the intrinsic death and birth rates respectively, leading to the net growth rate *r*_*R*_ = *β*_*R*_ − *δ*_*R*_. For simplicity, we assume that this stochastic extinction probability is a constant property of the resistant cell and does not depend on the control variable *u* directly (by assuming complete resistance) or indirectly via sensitive cell density (see Methods and materials). If control can also be exerted to the resistant cells (partial sensitivity), the optimal control can further capitalize on this establishment probability.

Now consider an *elimination strategy* $u:[0,T]\to U=[0,u_{\text{MTD}}]$, which gives the desired drug concentration over the treatment period. Here the end-time *T* is assumed fixed (see Materials and methods for more discussion) and *u*_MTD_ is the highest instantaneous drug concentration that can be delivered to the patient in question. Throughout, we assume that *u*_MTD_ ≤ *u*_max_, where *u*_max_ denotes the drug concentration where the pharmacodynamical effect is assumed to have plateaued. We make such a distinction, because it is important to separate the finite control leverage (*u*_max_) from the patient-specific constraint (*u*_MTD_) when using non-linear pharmacodynamics. For generality, we do not fix *u*_MTD_, but only *u*_max_, to allow meaningful comparisons between the MTD-strategy and the optimal treatment strategy.

With these assumptions, the intensity of the Poisson process, or the total cumulative rate of generating rescue mutations, is given by

$$\begin{array}{c} {\bar{n}}_{\text{rescue}}\left(u\right)=\int_{0}^{T}S\left(t,u\left(t\right)\right)\mu \left(u\left(t\right)\right)\pi_{f}dt, \end{array}$$ (1)

where *π*_*f*_ = 1 − *q* is the probability of establishment of a new mutant. This quantity corresponds to the expected number of successfully established rescue mutants generated during the treatment period. The probability of an evolutionary rescue by *de novo* mutation is then [2]

$$\begin{array}{c} 1-e^{-{\bar{n}}_{\text{rescue}}\left(u\right)}. \end{array}$$ (2)

The exponential term is just the zero class of the Poisson distribution and hence the complement of this gives the probability that there is at least one cell which survived the rescue window. A suitable objective of the treatment is then to minimise this quantity, which is equivalent to maximising the extinction probability of the target population. Since the probability of establishment is here just a constant, the objective functional for the optimal control problem reduces to

$$\begin{array}{c} C\left(u\right)=\int_{0}^{T}S\left(t,u\left(t\right)\right)\mu \left(u\left(t\right)\right)dt,\;u\left(t\right)\in U, \end{array}$$ (3)

which corresponds to the expected number of mutant establishment attempts. The discussed control problem is then to find the optimal elimination strategy, which minimises the cost functional above from the space of all (Lebesque integrable) functions over the treatment period.

Because the rate of generating rescue mutations is proportional to the population size, a characteristic feature of the rescue window is that the probability of evolutionary rescue sharply decreases with decreasing population size. This phenomenon corresponds to the classical somatic mutation theory of drug resistance and justifies the MTD-strategy which aims to minimise the probability of an evolutionary rescue by making the rescue window as short as possible. Indeed, suppose that the phenotypic mutation rate *μ* is independent of the control variable. Then, the MTD-solution *u* ≡ *u*_MTD_ is trivially the optimal treatment strategy (assuming we have control leverage ∂*S*/∂*u* < 0) and the treatment can be optimised only with respect to the delivery mode that satisfies the cumulative toxicity constraint. However, if *μ*′(*u*)>0, then clearly the MTD-solution is generally not optimal, because now the population size can be decreased only at the expense of increasing the mutation rate leading to an interesting and potentially exploitable trade-off, where the optimisation can be done also with respect to the cumulative drug concentration.

**Fig 2:**
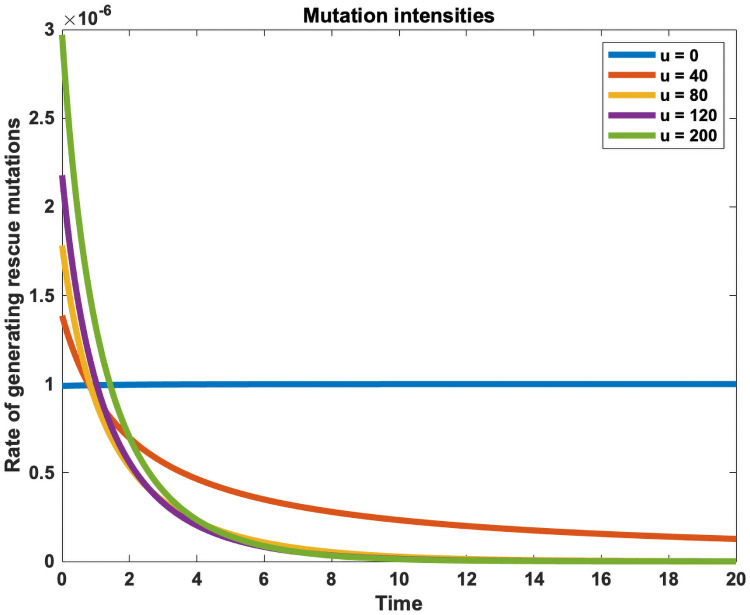
Time-dependent mutation rate profiles. Each treatment strategy *u*(*t*) leads to a characteristic mutation intensity profile *S*(*t*, *u*(*t*))*μ*(*u*(*t*)), which gives the rate of gaining a rescue mutant as a function of time. If no treatment is administered (*u*(*t*) = 0), the population grows to its carrying capacity and generates rescue mutations at a constant baseline mutation rate (blue). A dose-dependent mutation rate introduces a trade-off, where treatment can be used to decrease the population size only at the expense of simultaneously increasing the mutation rate. This creates a sharp mutation peak at the beginning, when treatment is applied to a large population size. The optimal treatment strategy, which minimises the probability of evolutionary rescue, exploits this trade-off by balancing the early mutational peak such that the area under intensity profile is minimised. The plotted intensity profiles were generated using the constant doses given in the legend and the same model and parameters as given in the Methods section (Eq 7 and Table 1).

Fig 2 shows an example of the intensity at which rescue mutations are generated during treatment. If no treatment is administered (*u* = 0), the population grows to its carrying capacity and generates rescue mutants with the constant baseline mutation rate *μ*_0_. When treatment is administered (*u*(*t*)>0), the mutation probability sharply decreases as the population size decreases. However, the drug-induced effects create a mutational peak in the beginning, where the probability of rescue mutations rises above the baseline mutation rate as control is being applied to a large population size. Higher doses lead to a higher early mutational peak, but the probability of rescue mutations decreases faster as the sensitive cell population diminishes faster than at lower doses. Lower doses on the other hand have a lower early mutational peak, but as it takes longer to eliminate the sensitive cells, the mutation probability decreases more slowly, thus prolonging the rescue window. The optimal strategy, which minimises the total cumulative rate of generating rescue mutations (or the area under the intensity profile), is a trade-off between these opposing treatment effects.

For evolutionary rescue to occur, the time at which the rescue mutation emerges plays no role. Early and late mutations are considered equally bad if the clinical objective is to maximise the probability of a complete cure. However, if evolutionary rescue does occur then the time of mutation is integral in determining the expected rescue fraction. This is simply because resistant cells that emerge early during the treatment period can generate much more growth than resistant cells that occur late. To minimise the expected number of resistant cells at the end of the treatment period, we need to weigh each mutation by the growth it can generate. Assuming a simple exponential growth of the resistant cell population at rate *r*_*R*_, the cost functional needs to be modified with a discount term $e^{r_{R}\left(T-t\right)}$, which equals to the growth generated by a resistant cell that emerged at time *t*. We refer to the problem

$$\begin{array}{c} C_{\text{discounted}}\left(u\right)=\int_{0}^{T}S\left(t,u\left(t\right)\right)\mu \left(u\left(t\right)\right)e^{r_{R}\left(T-t\right)}dt,\;u\left(t\right)\in U. \end{array}$$ (4)

as the discounted problem and show that the strategy, which minimises the expected number of resistant cells at the end, uses even lower doses to further reduce the early mutational peak.

### Intermediate dosages become optimal already with modest dose-dependency

When the perturbed growth dynamics and the dose-dependent mutation rate are specified, the optimal treatment strategy, which minimises the chosen objective, can be calculated using the methods of optimal control theory. As the cost functional (3) depends only on the sensitive cells (and there are initially so few resistant cells present that their competitive effect on the sensitive cells is negligible), we can use deterministic dynamics to calculate the mutation intensities. Using a logistic growth model with Hill-type pharmacodynamics and linear dose-dependent mutation rate (Eq (7), Materials and methods) we first solved the optimal control problem (3) using the Forward-Backward Sweep Method [36], which is based on Pontryagin’s minimum principle. The optimal control strategy *u*(*t*) together with the optimally controlled trajectories are shown in S1 Fig. Resistance will always emerge when using the deterministic dynamics due to the infinitesimal mutational flux generated during each time step. We thus further performed stochastic population dynamics simulations to gain a more realistic depiction of resistance evolution, as discussed later.

To gain further insights, we solved the same problem using an alternative approach based on the Hamilton-Jacobi-Bellman equation. The resulting control map *u*(*S*, *t*) (S2 Fig) is explicitly time-dependent only at the end of the treatment period, which is a boundary effect due to the fixed end-time. Therefore, the results are insensitive to the precise time implementation provided the end-time *T* is sufficiently large such that the sensitive cells can be eliminated during the treatment. In these cases, we can solve for a closed-loop control law *u*(*S*), which depends only on the current population size (S3 Fig). If the treatment period is shorter, the precise implementation time becomes important as the optimal treatment strategy will switch to use no control towards the end.

The time-independent control law can be derived analytically in an implicit form from the Hamilton-Jacobi-Bellman equation by requiring stationarity (see S1 Text). As we set the initial resistant cell population to zero and consider only elimination strategies, we can separate the sensitive and resistant dynamics so that the cost functional and the dynamics are independent of the number of resistant cells. For problem (3) with arbitrary density-dependent growth rate *r*(*S*), pharmacodynamics *d*(*u*) and dose-dependent mutation rate *μ*(*u*), we derive the following equation (see S1 Text)

$$\begin{array}{c} \mu \left(u\right)-[r(S)-d(u)-\mu (u)]\frac{\mu^{\prime }\left(u\right)}{d^{\prime }\left(u\right)+\mu^{\prime }\left(u\right)}=0, \end{array}$$ (5)

which can readily be solved for the control law *u*(*S*). Notice that here we assumed nothing about the precise functional form of the dose-dependent mutation rate *μ*(*u*), the pharmacodynamics *d*(*u*) or the density-dependent growth model *r*(*S*) except that these are all differentiable functions with respect to *u*. The only technical modelling assumption we have made is that of the *log-kill hypothesis*, where the control leverage depends only of the drug concentration and specifically does not depend on the population size. Therefore, the density dependence realises solely through the assumed density dependent growth rate *r*(*S*), which itself can be approximated to simple exponential decay during treatment. Hence, the optimal therapy is often close to a constant dose and lead us to compare simple constant treatment strategies.

Implementing the precise density and time dependencies lead only to marginal improvements and would be more difficult to realise clinically. However, the relative gain of the precise density and time implementation increases, when the drug is less effective (*d*_max_ is smaller) and when the pharmacodynamical profile is less steep (the Hill-coefficient is closer to 1). More generally, therapy optimisation with time-dependent protocol manipulations present a vast scope, e.g., an *in vitro* study with drug combinations demonstrate their ability to suppress, prevent or even reverse resistance against one of the drugs when a particular dosing is model is used [42]. New therapy strategies in this time dependent domain are being developed (see e.g. [43]), however, as all closed loop protocols hinge on continuous monitoring of the target cell population, formidable practical obstacles to their experimental testing remain [44].

**Fig 3:**
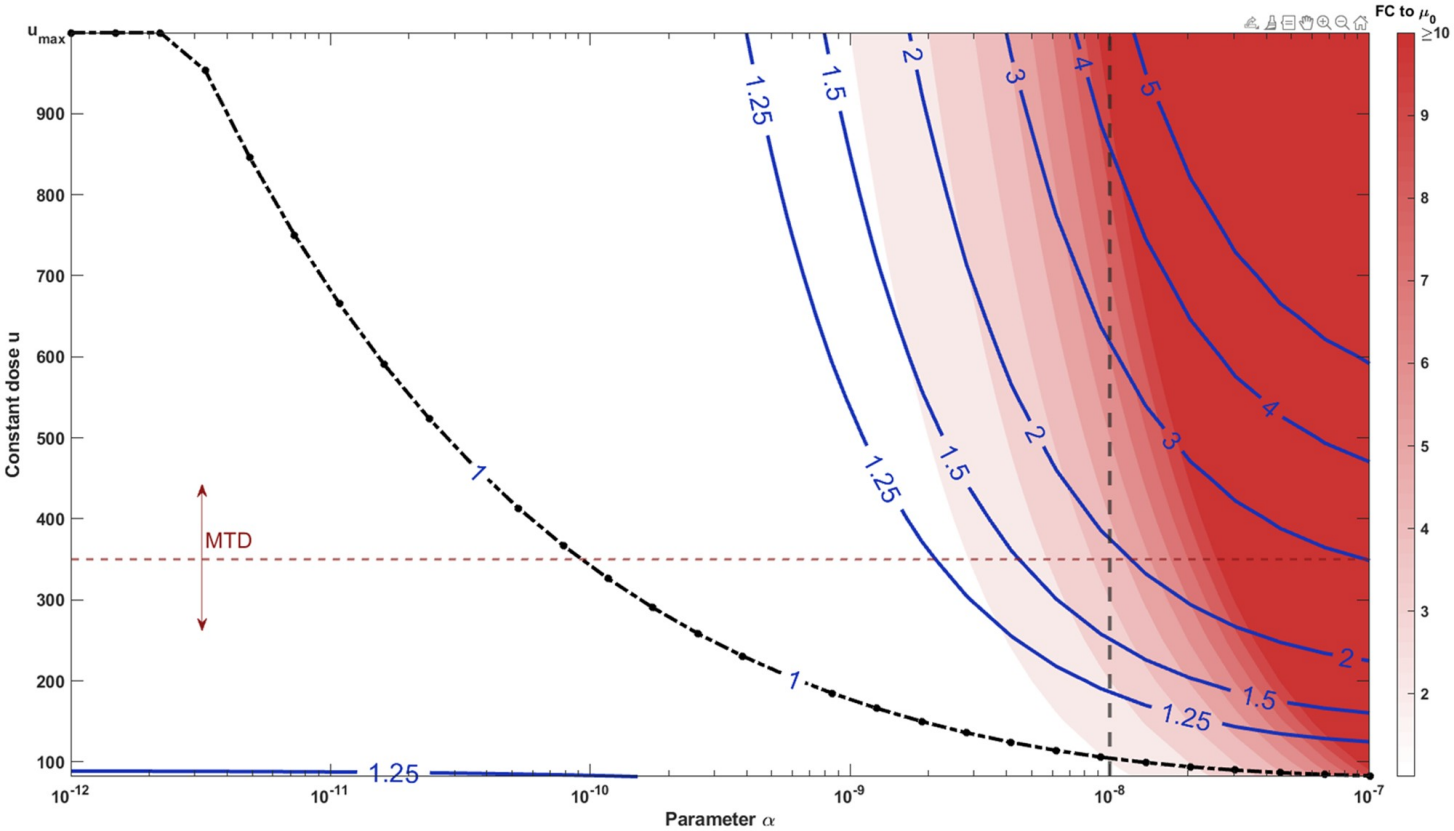
Optimal therapies substantially reduce the number of resistance mutations generated compared to MTD. The costs of constant therapies *C*(*u*;*α*) were evaluated while varying the key parameter *α*, which quantifies the strength of dose-dependent mutation rate. The plotted contour lines correspond to the cumulative mutation intensities (the expected number of rescue mutants) relative to the optimal constant treatment *C*(*u*;*α*)/*C*(u*;*α*). Thus, the 1-isoline (drawn as black dash-dot line for emphasis) gives the optimal constant dose as a function of the parameter *α*, while the 2-isoline gives the cases where the corresponding MTD produces 100% more rescue mutations than the optimal dose. The red background color indicates the assumed fold change (FC) to the baseline mutation rate. We notice that substantial improvements are possible even for modest fold-changes depending on how well the drug is tolerated (how close the MTD is to *u*_max_). The probability of evolutionary rescue scales exponentially in the amount of the rescue mutations. We consider the case *α* = 10^−8^ in detail, which corresponds to the cases given by the vertical dashed line. Similarly, the horizontal dashed line corresponds to the MTD used in stochastic simulations.

Substituting the linear dose-dependent mutation rate and Hill-type pharmacodynamics in Eq (5), we obtained the optimal elimination strategy as a function of the key parameter *α* (which is the slope of the assumed linear dose-dependent mutation rate) and compared its performance to other constant treatment strategies. Fig 3 displays the optimal constant treatment strategy u* (the black dash-dot line) in the relevant region of parameter *α*. Higher doses increase the cumulative mutation intensity or the expected number of rescue mutants generated. As different drugs have different toxicity constraints, there is no universal MTD to compare the optimal dose to. Hence, we regard the MTD as a variable and show few contour lines, where the labels denote the relative cumulative mutation intensity compared to the optimal. The red background color denotes the assumed fold change (FC) to the baseline mutation rate for corresponding *α* and constant dose.

We notice that the clinical gain of the optimal treatment depends heavily on the drug toxicity; the optimal treatment can lead to substantial gains with already modest drug-induced mutation if the drug is well-tolerated and administered close to, or at, the pharmacodynamical plateau (here *u*_max_ = 1000). On the other hand, no substantial gains are achievable even for a highly mutagenic drug if it is also poorly tolerated (i.e., MTD and optimal dosage are close to each other). Here we concentrated our analysis on the case *α* = 10^−8^, which leads to only modest fold-change of less than 10 (the vertical dashed line in Fig 3) when compared to the base mutation rate. Following the dashed line reveals that gains on the order of 25—100% are achievable already well below the maximum dose *u*_max_, which produces up to almost 5 times more rescue mutations than the optimal. We further note that the rescue probability scales exponentially in the amount of rescue mutations generated (see Eq (2)). Therefore, these differences are substantial at the probability level, whenever the baseline mutation intensity *Nμ*_0_ is relatively high but still mutation-limited. Indeed, in extreme cases, applying MTD in contrast to the optimal intermediate dosage will switch the emergence of resistance from a rare, mutation-limited, stochastic event to an inevitable outcome.

### Stochastic cell population model demonstrates the efficacy of intermediate dosage therapy under dose-dependent mutation

**Fig 4:**
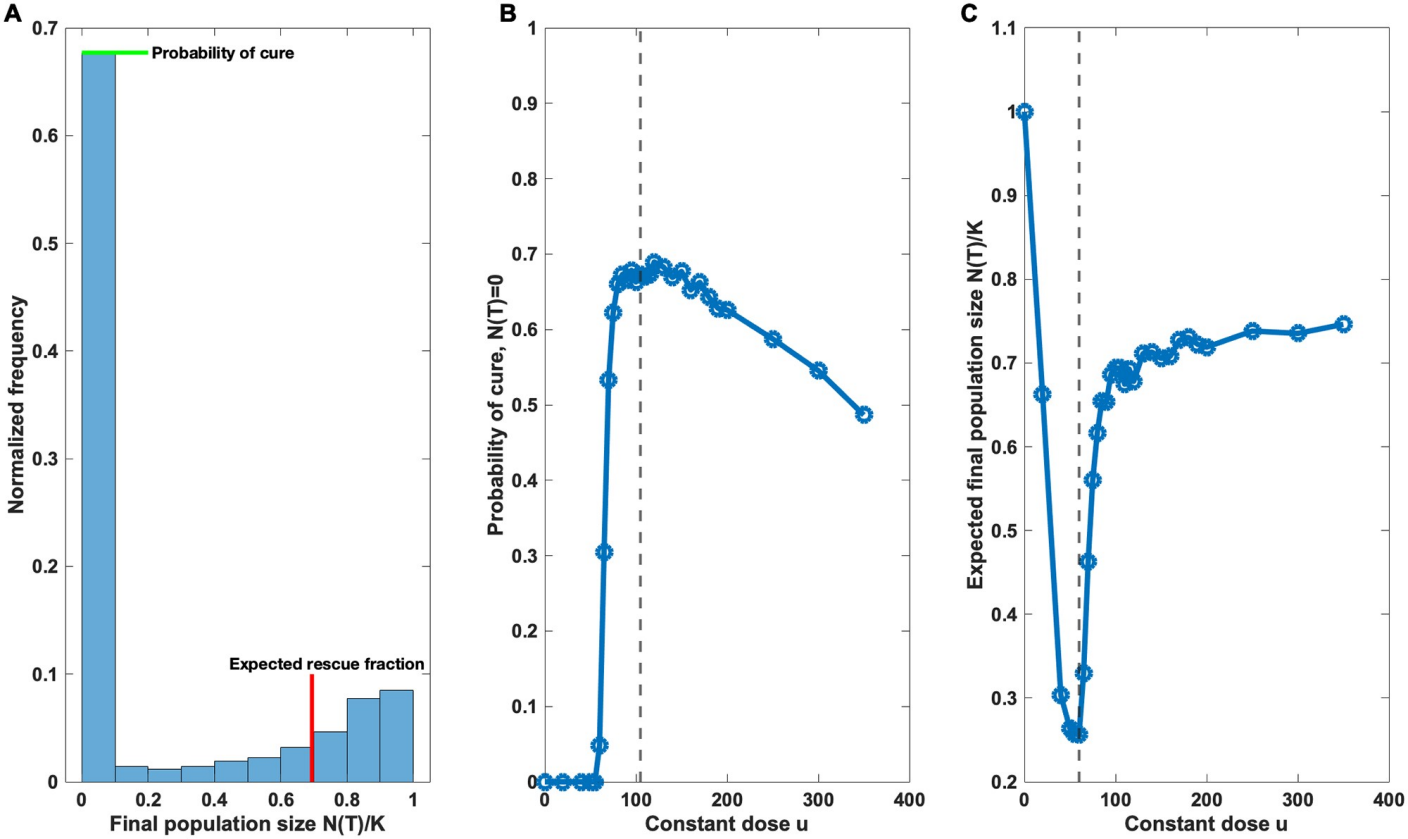
Non-monotonic dose responses. *n*_sim_ = 2000 constant therapies were simulated for each dose while recording the final population sizes *N*(*T*). **A** Example of a bimodal distribution of the normalized final population sizes *N*(*T*)/*K* using the optimal constant dose *u* = 104.5 that minimises the rescue probability (cost function Eq (3)). The zero mode corresponds to the proportion of extinct populations (cure) and the second mode corresponds to the expected size of the rescued population. Each dose leads to its own characteristic bimodal distribution. **B** The proportion of extinct populations *N*(*T*) = 0 plotted as a function of dose. The probability of cure displays non-monotonicity and is maximised in the neighborhood of the optimal dose *u* = 104.5 (dashed line), which was determined analytically using the stationary Eq (5). **C** The mean final population sizes of the rescued populations *N*(*T*)/*K* plotted as a function of dose (the extinct populations were excluded). The expected rescue size is minimised at *u* = 60 (dashed line), which agrees to the numerical solution S3 Fig of the discounted problem Eq (4).

To further validate our results we performed stochastic simulations and compared various constant therapies in terms of rate of successful eliminations and the size of rescued populations (Fig 4). The simulations revealed a characteristic bimodal distribution of the final population sizes after treatment, in which the first mode corresponds to the extinct populations and the second mode to the expected size of the rescued populations. The maximisation of the first mode corresponds to the cost function defined in Eq (3), where the probability of an evolutionary rescue is minimised, whereas the minimisation of the second mode corresponds to the discounted cost function Eq (4). Hence, both modes can be analyzed and optimised mathematically using the simpler deterministic dynamics discussed above.

Fig 4 summarizes the results obtained from stochastic simulation. Fig 4A displays the bimodality of the distribution of final population sizes. Each treatment strategy leads to its own characteristic bimodal distribution. Fig 4B shows how the zero mode, that is, the probability of cure, changes as a function of the dose. The different mutation intensities have a substantial impact on the probability of cure and we notice how therapies close to the optimal treatment strategy outperform and lead to substantially better expected treatment outcomes. Similarly Fig 4C shows an interesting non-monotonic dose response in the expected final population sizes of the target populations that survive from the therapy (the mean of normalized final population sizes *N*(*T*)/*K* conditioned on non-extinction).

The solution of the discounted problem uses even less control in the beginning to shift the expected mutation time to later time points. This of course comes at the expense of increasing the total cumulative mutation rate, and hence, decreases the probability of a cure. This might be acceptable if the probability of evolutionary rescue is in any case high. However, treatment attempting for a cure is always riskier in the sense that if evolutionary rescue does occur, the rescue mutations take likely place very early on thus leading to relapse more quickly. Therefore, it is the baseline expectation of the likelihood of evolutionary rescue which should ideally guide the treatment choice and the precise evolutionary objectives of the treatment.

We conducted the simulations by setting the baseline (drug-free) mutation intensity at the start of therapy to *S*_0_ *μ*_0_ = 0.1. If the baseline mutation intensity is close to 1 or higher, then evolutionary rescue occurs with very high probability in any case, and the relative role of the drug-induced mutations become less important. However, we would like to emphasize that the viability of *any* elimination strategy relies on mutation limitation, and in these cases, the drug-induced effects become crucially important, and the optimised treatments may lead to substantial improvements as demonstrated in Fig 4.

## Discussion

The evolution of drug resistance is a particularly problematic and frequent outcome of cancer and antimicrobial therapies. Recent research suggests that these treatments may enhance the evolvability of the target population not only via inducing intense selection pressures but also via increasing mutability, and thus the speed of adaptation. Although it remains unclear whether the observed increases in the mutation rate are simply an indirect physiological response to stress or rather an adaptive trait itself, such mutation rate plasticity has a clear implication for evolutionary theory: any stress-induced increase in mutation supply will necessarily (all else equal and barring mutational meltdown) increase the potential for resistance evolution and one can no longer isolate mutational processes from the underlying severity of selection.

Here we have analysed a model with drug-induced increase in mutation rate, which leads to an interesting trade-off, where the population size can be decreased only at the expense of simultaneously increasing the mutation rate. Using the concept of evolutionary rescue, we formulated the treatment as an optimal control problem and solved the optimal elimination strategy, which minimises the probability of evolutionary rescue. We showed using stochastic simulations that aggressive elimination strategies, which aim at eradication as fast as possible and which represent the current standard of care, can be detrimental already with modest drug-induced increases to the baseline (drug-free) mutation rate.

The assumed increases in the mutation rate are roughly the same order of magnitude as those reported in a recent meta-analysis focusing on common antibiotics in sub-inhibitory concentrations [45]. It seems likely that the drug-induced effects may be even more pronounced in cancer treatments given their known mutagenicity, and in clinically relevant concentrations above MIC which leads to rapid decay as assumed in our model. On the other hand, it is also probable that the required mutational targets change at higher concentration ranges, thus in turn decreasing the phenotypic mutation rate. Nonetheless, the basic conclusion of our work remains, namely that the mutation rate can in many clinically relevant settings be highly dose-dependent, and this in turn implies that the MTD-strategy is no longer necessarily optimal. Therefore, it will be of great importance to properly investigate the various mutagenic and other resistance-promoting properties of different anticancer and antimicrobial therapies across wide concentration ranges to recover the drug-specific mutation rate profile.

Our main focus was to solve the optimal elimination strategy, which minimises the probability of an evolutionary rescue, and the optimal solution thus reduces to the MTD-strategy in the limit of dose-indepedent mutation rate (*α* → 0). Recently, however, the objective of eliminating the tumor burden has been challenged and so-called containment strategies have been proposed to specifically avoid the competitive release of the resistant cells. Such paradigm shift in treatment may greatly improve treatment outcomes especially in those situations where there is high abundance of pre-existing resistant cells and a complete cure cannot be expected. First proofs of concept have already been made by Gatenby and colleagues in preclinical mouse models and advanced metastatic cancers [46, 47] and recently also in the context of antimicrobial resistance [35]. Based on these findings, Hansen *et al*.[35] argue that all viable treatment strategies must trade-off between minimising mutations (to prevent the emergence of new resistant cells) and maximising competition (to suppress the growth of the existing resistant cells).

**Fig 5:**
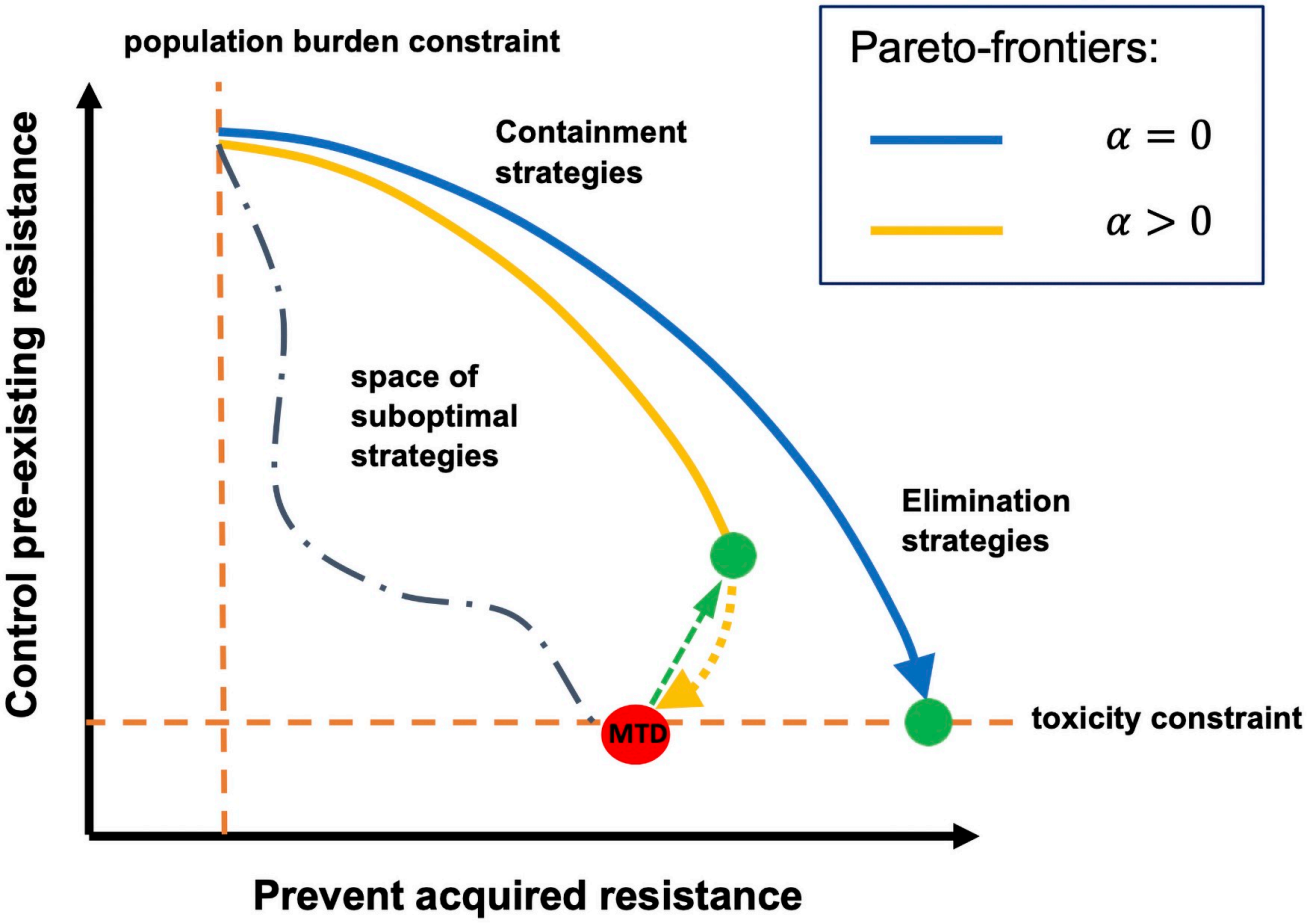
Trade-offs in treatment optimisation. Every treatment strategy is necessarily a trade-off between preventing acquired resistance, by decreasing the population size, and suppressing pre-existing resistance, by allowing intercellular competition. The rate at which the population size can be decreased is constrained from above by the toxicity constraint as well as by finiteness of control leverage and, on the other hand, from below by the population burden constraint, which forces to apply control to stabilize the population size at some acceptable level. When no drug-induced effects are present (*α* = 0), the optimal treatment strategy is found somewhere on the blue Pareto-frontier; the arrow points to the direction where the cumulative drug concentration increases and the optimal elimination strategy (the green point) is given by the MTD-strategy. However, if drug-induced effects are present (*α* > 0), the optimisation must be done on a completely different, yellow Pareto-frontier, which exhibits a bifurcation point after which increasing the cumulative drug concentration becomes detrimental with both respects. In these cases, the optimal elimination strategy (the green point) is reached at intermediate dosages at the bifurcation point, which can be identified using the methods presented here. Hence, substantial Pareto-improvements (represented by the green arrow) may be achieved by switching from the MTD-strategy to the optimised treatments.

To put our results into a wider context, consider Fig 5 which illustrates this fundamental trade-off between the two alternative evolutionary pressures that can be induced by treatment. So far, the discussion of treatment optimisation has exclusively concentrated on the blue trade-off curve, in effect assuming that MTD-strategies minimise the expected number of rescue mutations. The key result obtained here is that this is not generally true when there are drug-induced effects present as the optimisation must then be performed on a completely different trade-off curve. Neglecting the effects of drug-induced resistance can lead to situations where the MTD-strategy lies well below the correct trade-off curve and is thus a particularly detrimental strategy as it fails to optimally prevent acquired resistance by *de novo* mutation, but also applies maximal damage to the ecosystem making the containment of pre-existing resistant cells impossible.

Using the methods presented here, one can identify the optimal mutation-minimising solution and thus potentially gain substantial improvements. Furthermore, the insights gained while studying the discounted problem may be useful also in the context of containment strategies, where partial elimination is sought while lowering the tumor or pathogen burden to an acceptable level. Thus, when the clinical objective shifts from cure to resistance management, an initial elimination strategy which minimises the expected number of resistant cells becomes a rational objective. While it is well-known that attempting a cure may be riskier in terms of expediting progression because of standing variation [48], the discounted problem shows that this could be the case also when relapse originates by *de novo* mutation.

For the case of AMR evolution, our result that intermediate dosage therapy is optimal is particularly interesting as it may also reduce detrimental off-target species effects, such as enriching for resistance in off-target species [12] or compromising community resilience and functioning [49]. Such effects are prevalent in bacterial communities and possibly important to the AMR problem as a whole [12]. As stated above, intermediate dosages may also be optimal in containment strategies [35], which may be useful in chronic infections prevalent owing to factors such as antimicrobial tolerance [23] and biofilms [50] as features of the pathogen population and immunocompromised conditions in the patient. Our findings therefore contribute to an emerging body of evidence showing an increasing scope of utility for intermediate dosages in antimicrobial therapy. Drug-induced mutagenesis has also been observed for viruses [51]. Although the pharmacodynamics differ considerably from cellular systems, some of our observations may also extend to viruses, at least the observation that other concentrations than the MTD may lead to better therapy outcomes when mutation rate effects of drugs are accounted.

The approach taken here has many advantages. We presented a way of formulating the precise objectives of the treatment in evolutionary terms, which provides an interesting theoretical framework for further treatment optimisation avenues. We specifically considered the effects of drug-induced resistance, an often neglected cost of treatment, and showed that reducing the cumulative drug dosage can be preferable even when the objective of the treatment is elimination, and not containment. Therefore, our results further add to the ongoing criticism of administering aggressive, high-dose therapies, which may realize not only an ecological, but also an evolutionary cost of control.

The predicted non-monotonic dose responses of the target populations are also immediately amenable to experimental investigation, where a lot of future study is needed to gain a better quantitative knowledge of the modes and extent of drug-induced effects. Drugs with reported increases in mutation rate present a good starting point (see e.g. [15, 16, 45]). Our work makes concrete testable predictions, which potentially allows to robustly detect the presence of drug-induced resistance evolution without a need to try measure the actual mutation rate directly [52], but instead focusing on the dose-dependency in the observed rescue probabilities. If indeed such phenomena increase the rate of adaptation, we should see similar non-monotonic dose responses in the rescue probabilities as in Fig 4, a result that could be exploited in therapy optimisation. Fully disentangling the individual terms behind such a dose response, i.e. intrinsic mutation rate, effects of varying mutational targets, phenotypic plasticity and the role of selection and establishment, would require more detailed modelling, e.g., a stochastic treatment of the resistance mutations below their establishment threshold.

When interpreting the more quantitative predictions made by our work, care must be taken, as they naturally depend on parameter values and more implicit modelling choices (see Materials and methods for more discussion). For instance, this is the case for the linear dose-dependent mutation rate, where we currently lack extensive data on the dose dependency. However, since nothing in the derivation of Eq (5) hinges on this particular choice, the provided analytical approach can be used to generalize our results to a wide class of models and empirically obtained functional forms.

**Fig 6:**
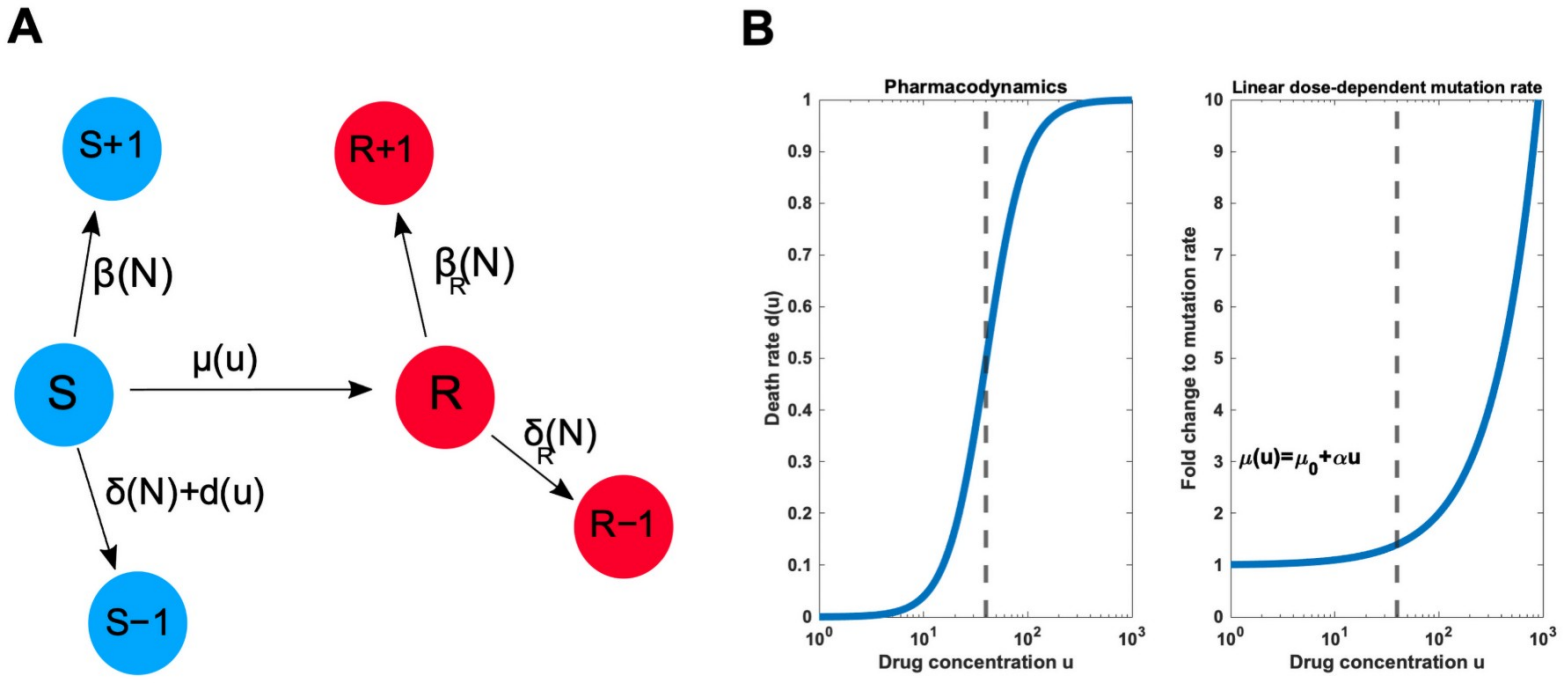
Schematic illustration of the model. **A** A minimal model for drug resistance distinguishes sensitive (*S*) and resistant (*R*) cells, which follow their own birth-death processes. A treatment can be used to target sensitive cells, but sensitive cells can become resistant via rescue mutations. **B** Specification of dose-dependent death and mutation rates. We analyse a Hill-type pharmacondynamics and linear dose-dependent mutation rate, but any pharmacodynamics with finite control leverage and monotonically increasing mutation rate will lead to qualitatively similar results reported here. The dashed lines denote the growth inhibitory drug concentration.

Another important modelling assumption of our work is that the mutation rates are not strictly coupled to birth events (see Fig 6), which may explain why Liu *et al.* [37] did not find the dose-dependent mutation rates important in the context of cytostatic treatments. This is because then one actually assumes that the therapy effectively prevents any mutations from accumulating. There are several reasons why we think that this, otherwise common modelling choice, is a poor assumption in the context of treatment models. Firstly, the mutation rate term in our model should not be interpreted as just the genomic substitution rate (which only rarely actually lead to a more adaptive phenotype), but rather as the Poisson intensity of generating resistant phenotypes in a given environment. While the majority of spontaneous genomic mutations do indeed occur during replication, there is no underlying reason why specific drug-induced mutations and non-genetic phenotype transitions could not also happen in other phases of the cell cycle. Indeed, recent evidence from single cell sequencing shows that neurons accumulate somatic mutations at a constant rate throughout life without cell division, with similar rates to mitotically active tissues [53]. Even if the considered phenotypic transitions occurred proportional to the cell turnover, it would be an unwarranted simplification to assume that even purely cytostatic treatments would completely halt cell division in the entire cell population. If this, however, would be the case, then evolutionary rescue could occur only via standing variation, which again shifts the focus of treatment optimization towards containment strategies (if one has no direct control leverage over the resistant cells).

The purpose of our work was to analyse a minimal model to study the effect of drug-induced resistance evolution instead of providing the most realistic and comprehensive description of the underlying processes. The provided optimal control approach can be used to generalise our results to a wide class of models, once we have a better quantitative understanding of the various dose-dependencies involved. Given the wide-spread use of aggressive, MTD-style therapies, these results may be important and worth of further investigation even if they only apply to certain drugs. In any case, we believe that identifying and factoring into therapy optimisation the various, as of yet unknown or unquantified, biological costs of therapeutic control represent a major goal and unifying principle going forward.

## Methods and materials

### Dynamical model for drug resistance

Consider the problem of finding the optimal elimination strategy that minimises the probability of evolutionary rescue by *de novo* mutation in the case of drug-induced resistance. First, consider the following general dynamical model for drug resistance:

$$\begin{array}{c} \left\{ \begin{array}{cc} \dot{S} & =r_{S}\left(N\right)S-d_{S}\left(u,N\right)S-\mu_{S}\left(u\right)S+\mu_{R}\left(u\right)R\\ \dot{R} & =r_{R}\left(N\right)R-d_{R}\left(u,N\right)R+\mu_{S}\left(u\right)S-\mu_{R}\left(u\right)R \end{array}\right. \end{array}$$ (6)

*S*(*t*) and *R*(*t*) are the state variables denoting the population densities for the sensitive and resistant cells, respectively. The functions *r*_*S*_(*N*) and *r*_*R*_(*N*) are the unperturbed growth rates at which sensitive and resistant cells, respectively, grow in the absence of treatment. The growth rate can be different for the sensitive and resistant cells, for example, due to the fitness cost that results from maintaining the resistance mechanism. Constant growth rates lead to the exponential growth model (which is suitable only for small populations) while common density-dependent choices, which depend on the total population size *N*(*t*) ≔ *S*(*t*) + *R*(*t*) via competitive interactions, include logistic $r_{i}(N)=r_{i}(1-\frac{N}{K})$ and Gompertz $r_{i}(N)=r_{i}\text{log}(\frac{K}{N})$ growth models, where *K* is the assumed common carrying capacity and *i* ∈ {*S*, *R*}.

The function *d*_*i*_(*u*, *N*) models the pharmacodynamics of the drug dictating how the obtained concentration of the drug, which is represented by the control variable *u*, translates into cell death. Some drugs can also be cytostatic in nature, meaning that they decrease the birth rate instead of increasing the death rate, which can have important consequences [54] but nevertheless leads to the same mean-field growth as above. Finally, the pharmacodynamical effect may additionally depend on the total population size *N*. By definition, we concentrate on cases where *d*_*S*_(*u*, *N*) ≫ *d*_*R*_(*u*, *N*) ≥ 0 meaning that resistant cells have a selective advantage during treatment.

Finally, the function *μ*_*S*_(*u*) is the rate at which sensitive cells become resistant. Importantly, we allow this rate, too, to explicitly depend on the dosage *u*. Furthermore, note that we do not distinguish between the precise cause (genetic and non-genetic) of the change in phenotype, but only consider the transition between the two compartments. Reversible adaptive (epigenetic) changes can be modelled by adjusting the *μ*_*R*_ term.

To demonstrate the qualitative impact of the dose-dependent mutation rate, consider the following simple model with logistic growth, Hill-type pharmacodynamics and linear dose dependency

$$\begin{array}{c} \left\{ \begin{array}{cc} \dot{S} & =r_{S}\left(1-S-R\right)S-d_{\text{max}}(1-\frac{1}{1+{\left(\frac{u}{h}\right)}^{k}})S\\ & \;-\left(\mu_{0}+\alpha u\right)S;\;S\left(0\right)=1\\ \dot{R} & =r_{R}\left(1-S-R\right)R+\left(\mu_{0}+\alpha u\right)S;\;R\left(0\right)=0. \end{array}\right. \end{array}$$ (7)

This model follows closely the scaled dynamical model (i.e. *K* = 1) used by Greene *et al.* [38] but includes more realistic non-linear pharmacodynamics. Now the cost function of Eq (3) can be minimised with respect to the dynamics (7) with two alternative methods based either on Pontryagin’s Minimum Principle (see e.g [34, 55]) or the Hamilton-Jacobi-Bellman (HJB) equation (see e.g. [44, 55]).

Pontryagin’s minimum principle leads to a system of ordinary differential equations that must be solved with mixed boundary conditions, for example, by using the Forward-Backward Sweep Method [36]. The HJB approach on the other hand requires the solution of a partial differential equation for the cost-to-go function, which comes at a higher computational cost. We solve the optimal control problem using both these methods and furthermore provide some analytical insights to the optimal treatment strategy using the stationary profile obtained from the HJB solution (see S1 Text for further details).

### Stochastic simulation

To further demonstrate the qualitative impact of the dose-dependency, we performed a stochastic simulation of different constant therapies ranging from low to high concentrations. Each dosage yields its characteristic intensity profile at which rescue mutants are being generated. We show how this connects to the mutational profile and the resulting distribution of rescue fractions that survive treatment.

The stochastic simulation consists of simulating the system given in Eq (7) for a range of constant doses and different initial conditions *S*_0_, corresponding to different effective baseline mutation rates. For each dose, *n*_sim_ = 2000 virtual treatments were administered while recording mutation events and final numbers of sensitive and resistant cells. Unlike the deterministic system, the stochastic birth and death process allows the population to go extinct (a cure). By calculating the proportion of extinct populations for each dose we can estimate the probability of evolutionary rescue and its dose-dependency. Furthermore, by recording the mutational events and stochastic extinctions, we could verify that the resistance establishment probability is indeed approximately independent of the dose as assumed in cost function of Eq (3).

For the stochastic system, we need to explicitly specify the birth and death rates and how the carrying capacity is realized (parameter *θ*) [56]. The event propensities for the Gillespie algorithm are

$$\begin{array}{c} \left\{ \begin{array}{cc} \beta_{S} & =(b_{S}-\left(b_{S}-\theta_{S}\right)\left(S+R\right)/K)S\\ \beta_{R} & =(b_{R}-\left(b_{R}-\theta_{R}\right)\left(S+R\right)/K)R\\ \delta_{S} & =(d_{S}+\left(\theta_{S}-d_{S}\right)\left(S+R\right)/K+d\left(u\right))S\\ \delta_{R} & =(d_{R}+\left(\theta_{R}-d_{R}\right)\left(S+R\right)/K)R\\ \mu & =(\mu_{0}+\alpha u)S \end{array}\right. \end{array}$$ (8)

where the events are defined as

$$\begin{array}{c} \left\{ \begin{array}{cc} S & \overset{\beta_{S}}{\to }S+1\\ S & \overset{\delta_{S}}{\to }S-1\\ R & \overset{\beta_{R}}{\to }R+1\\ R & \overset{\delta_{R}}{\to }R-1\\ S,R & \overset{\mu }{\to }S-1,R+1. \end{array}\right. \end{array}$$ (9)

Parameter values that were used in this study are given below in Table 1. We note that the key parameter *α* that sets the drug-induced mutation rate slope was selected so that the dose-dependent mutation rate covers an order of magnitude (see Fig 3 and e.g. [15]). The key elements to observe the discussed trade-off are finiteness of control leverage (with molecular binding based control this is generally true) and monotonically increasing dose to intrinsic mutation rate dependency. The other parameters are chosen such that, the therapy can enforce the sensitive population to decay while the resistant cell can grow once established. These rates then set a sufficient time *T* that ensures that in most cases the sensitive population has been eradicated and the resistant population, if established, occupies a substantial part of the released niche. In practice, the end-time is sufficiently long if the control map obtained from the HJB solution becomes stationary (see example S2 Fig). As discussed earlier the product *S*_0_ *μ*_0_ fixes to what degree the evolution of resistance is mutation limited. If that product is large to begin with there is not much help in optimising the therapy-induced mutations. In such case the objective of the treatment should move away from eradication.

**Table 1:** Table of parameters used. ([*t*] = unit of time, [*u*] = unit of drug concentration).

Symbol:	Meaning:	Value:
*μ*_0_	baseline mutation rate	10^−6^/[*t*]
*α*	slope coefficient of *μ*(*u*)	10^−8^/([*u*] ⋅ [*t*])
*b*_*S*_	intrinsic birth rate of sensitive cells	0.8/[*t*]
*d*_*S*_	intrinsic death rate of sensitive cells	0.3/[*t*]
*r*_*S*_	intrinsic growth rate (*β*_*S*_ − *δ*_*S*_) of sensitive cells	0.5/[*t*]
*b*_*R*_	intrinsic birth rate of resistant cells	0.5/[*t*]
*d*_*R*_	intrinsic death rate of resistant cells	0.1/[*t*]
*r*_*R*_	intrinsic growth rate (*β*_*R*_ − *δ*_*R*_) of resistant cells	0.4/[*t*]
*d*_max_	maximum death rate by treatment	1.0/[*t*]
*u*_max_	drug concentration where the death rate has saturated	10^3^ ⋅ [*u*]
*k*	Hill-coefficient of *d*(*u*)	2.3
*h*	drug concentration yielding 50% of *d*_*max*_	40 ⋅ [*u*]
*T*	fixed end-time of treatment cycle	35 ⋅ [*t*]
*K*	carrying capacity	10^6^
*S*_0_	initial population size of sensitive cells	10^5^
*R*_0_	initial population size of resistant cells	0
*θ*_*S*_	carrying capacity parameter for sensitive cells	0.8 ⋅ [*t*]
*θ*_*R*_	carrying capacity parameter for resistant cells	0.5 ⋅ [*t*]

## Supporting information

S1 Fig Numerical solutions using Forward-Backward Sweep Method. Solution of the optimal control problem (3) using Forward-Backward Sweep Method with parameter values specified in Table 1 (main text). **A** Optimal treatment strategy *u*_opt_(*t*) as a function of time. **B** The optimally controlled trajectories *S*(*t*) and *R*(*t*). In deterministic dynamics the population always experiences an evolutionary rescue. **C** The dynamics of the multipliers *λ*_*S*_(*t*) and *λ*_*R*_(*t*) corresponding to the sensitive and resistant cells respectively. The multiplier values can be interpreted as sensitivities of the optimal cost *C*(*u*_opt_) to the perturbations in the respective state variables.
(TIF)

S2 Fig Control map obtained from the stochastic Hamilton-Jacobi-Bellman approach. Here the color denotes the optimal control to be applied at the given population size and time. The carrying capacity has been scaled to *K* = 100. Notice, how the control values start to change in time only at the end of control period, when *t* > 20.
(TIF)

S3 Fig Time-independent control laws. Feedback controls *u*(*S*) for the two optimal control problems obtained via the inverse function method. The dashed lines give the optimal constant doses, respectively. The analytically derived stationary profile matches the numerical solution, but cannot be applied to the discounted problem due to the explicit time-dependence.
(TIF)

S1 Text Extended mathematical description of the model and the related analyses. (PDF)

## References

[1] Gonzalez A, Ronce O, Ferriere R, Hochberg ME. Evolutionary rescue: An emerging focus at the intersection between ecology and evolution. Philosophical Transactions of the Royal Society B: Biological Sciences. 2013;368 (1610). doi: 10.1098/rstb.2012.0404 PMC3538460 23209175

[2] Alexander HK, Martin G, Martin OY, Bonhoeffer S. Evolutionary rescue: Linking theory for conservation and medicine. Evolutionary Applications. 2014;7(10):1161–1179. doi: 10.1111/eva.12221 25558278 PMC4275089

[3] Anciaux Y, Chevin LM, Ronce O, Martin G. Evolutionary rescue over a fitness landscape. Genetics. 2018;209(1):265–279. doi: 10.1534/genetics.118.300908 29535150 PMC5937192

[4] Read AF, Day T, Huijben S. The evolution of drug resistance and the curious orthodoxy of aggressive chemotherapy. Proceedings of the National Academy of Sciences of the United States of America. 2011;108(SUPPL. 2):10871–10877. doi: 10.1073/pnas.1100299108 21690376 PMC3131826

[5] Goldie JH, Coldman AJ. The Genetic Origin of Drug Resistance in Neoplasms: Implications for Systemic Therapy. Cancer Research. 1984;44(9):3643–3653. 6744284

[6] Benzekry S, Pasquier E, Barbolosi D, Lacarelle B, Barlési F, André N, et al. Metronomic reloaded: Theoretical models bringing chemotherapy into the era of precision medicine. Seminars in Cancer Biology. 2015;35:53–61. doi: 10.1016/j.semcancer.2015.09.002 26361213

[7] Day T, Huijben S, Read AF. Is selection relevant in the evolutionary emergence of drug resistance? Trends in Microbiology. 2015;23(3):126–133. doi: 10.1016/j.tim.2015.01.005 25680587 PMC4494118

[8] Martin RB, Fisher ME, Minchin RF, Teo KL. Optimal control of tumor size used to maximize survival time when cells are resistant to chemotherapy. Mathematical Biosciences. 1992;110(2):201–219. doi: 10.1016/0025-5564(92)90039-Y 1498450

[9] Gatenby RA, Silva AS, Gillies RJ, Frieden BR. Adaptive therapy. Cancer Research. 2009;69(11):4894–4903. doi: 10.1158/0008-5472.CAN-08-3658 19487300 PMC3728826

[10] Monro HC, Gaffney EA. Modelling chemotherapy resistance in palliation and failed cure. Journal of Theoretical Biology. 2009;257(2):292–302. doi: 10.1016/j.jtbi.2008.12.006 19135065

[11] Hansen E, Woods RJ, Read AF. How to Use a Chemotherapeutic Agent When Resistance to It Threatens the Patient. PLoS Biology. 2017;15(2):1–21. doi: 10.1371/journal.pbio.2001110 28182734 PMC5300106

[12] Morley VJ, Woods RJ, Read AF. Bystander Selection for Antimicrobial Resistance: Implications for Patient Health. Trends in Microbiology. 2019;27(10):864–877. doi: 10.1016/j.tim.2019.06.004 31288975 PMC7079199

[13] Luria SE, Delbrück M. Mutations of Bacteria from Virus Sensitivity to Virus Resistance. Genetics. 1943;28(6):491–511. doi: 10.1093/genetics/28.6.491 17247100 PMC1209226

[14] Fitzgerald DM, Rosenberg SM. What is mutation? A chapter in the series: How microbes “jeopardize” the modern synthesis. PLoS genetics. 2019;15(4):e1007995. doi: 10.1371/journal.pgen.1007995 30933985 PMC6443146

[15] Long H, Miller SF, Strauss C, Zhao C, Cheng L, Ye Z, et al. Antibiotic treatment enhances the genome-wide mutation rate of target cells. Proceedings of the National Academy of Sciences of the United States of America. 2016;113(18):E2498–E2505. doi: 10.1073/pnas.1601208113 27091991 PMC4983809

[16] Russo M, Crisafulli G, Sogari A, Reilly NM, Arena S, Lamba S, et al. Adaptive mutability of colorectal cancers in response to targeted therapies. Science. 2019;366(December):1473–1480. doi: 10.1126/science.aav4474 31699882

[17] Cipponi A, Goode DL, Bedo J, McCabe MJ, Pajic M, Croucher DR, et al. MTOR signaling orchestrates stress-induced mutagenesis, facilitating adaptive evolution in cancer. Science (New York, NY). 2020;368(6495):1127–1131. doi: 10.1126/science.aau8768 32499442

[18] Kohanski MA, DePristo MA, Collins JJ. Sublethal Antibiotic Treatment Leads to Multidrug Resistance via Radical-Induced Mutagenesis. Molecular Cell. 2010;37(3):311–320. doi: 10.1016/j.molcel.2010.01.003 20159551 PMC2840266

[19] Krašovec R, Richards H, Gifford DR, Hatcher C, Faulkner KJ, Belavkin RV, et al. Spontaneous mutation rate is a plastic trait associated with population density across domains of life. PLoS biology. 2017;15(8):e2002731. doi: 10.1371/journal.pbio.2002731 28837573 PMC5570273

[20] Pich O, Muiños F, Lolkema MP, Steeghs N, Gonzalez-Perez A, Lopez-Bigas N. The mutational footprints of cancer therapies. Nature Genetics. 2019;51(12):1732–1740. doi: 10.1038/s41588-019-0525-5 31740835 PMC6887544

[21] Pisco AO, Brock A, Zhou J, Moor A, Mojtahedi M, Jackson D, et al. Non-Darwinian dynamics in therapy-induced cancer drug resistance. Nature Communications. 2013;4:1–11. doi: 10.1038/ncomms3467 24045430 PMC4657953

[22] Aktipis CA, Boddy AM, Gatenby RA, Brown JS, Maley CC. Life history trade-offs in cancer evolution. Nature Reviews Cancer. 2013;13(12):883–892. doi: 10.1038/nrc3606 24213474 PMC4010142

[23] Balaban NQ, Helaine S, Lewis K, Ackermann M, Aldridge B, Andersson DI, et al. Definitions and guidelines for research on antibiotic persistence. Nature Reviews Microbiology. 2019;17(7):441–448. doi: 10.1038/s41579-019-0196-3 30980069 PMC7136161

[24] Boumahdi S, de Sauvage FJ. The great escape: tumour cell plasticity in resistance to targeted therapy. Nature Reviews Drug Discovery. 2020;19(1):39–56. doi: 10.1038/s41573-019-0044-1 31601994

[25] Wood KB, Cluzel P. Trade-offs between drug toxicity and benefit in the multi-antibiotic resistance system underlie optimal growth of E. coli. BMC Systems Biology. 2012;6. doi: 10.1186/1752-0509-6-48 22631053 PMC3462682

[26] El Meouche I, Siu Y, Dunlop MJ. Stochastic expression of a multiple antibiotic resistance activator confers transient resistance in single cells. Scientific Reports. 2016;6(January):1–9. doi: 10.1038/srep19538 26758525 PMC4725842

[27] El Meouche I, Dunlop MJ. Heterogeneity in efflux pump expression predisposes antibiotic-resistant cells to mutation. Science. 2018;362(6415):686–690. doi: 10.1126/science.aar7981 30409883 PMC6343669

[28] Maltas J, Wood KB. Pervasive and diverse collateral sensitivity profiles inform optimal strategies to limit antibiotic resistance. vol. 17; 2019. 10.1371/journal.pbio.3000515 PMC6834293 31652256

[29] Roemhild R, Gokhale CS, Dirksen P, Blake C, Rosenstiel P, Traulsen A, et al. Cellular hysteresis as a principle to maximize the efficacy of antibiotic therapy. Proceedings of the National Academy of Sciences of the United States of America. 2018;115(39):9767–9772. doi: 10.1073/pnas.1810004115 30209218 PMC6166819

[30] Martinez JL, Baquero F. Mutation frequencies and antibiotic resistance. Antimicrobial Agents and Chemotherapy. 2000;44(7):1771–1777. doi: 10.1128/aac.44.7.1771-1777.2000 10858329 PMC89960

[31] Pérez-Velázquez J, Rejniak KA. Drug-Induced Resistance in Micrometastases: Analysis of Spatio-Temporal Cell Lineages. Frontiers in Physiology. 2020;11(April):1–12. doi: 10.3389/fphys.2020.00319 32362836 PMC7180185

[32] Lässig M, Mustonen V. Eco-evolutionary control of pathogens. Proceedings of the National Academy of Sciences. 2020;117(33):19694–19704. doi: 10.1073/pnas.1920263117 PMC7443876 32737164

[33] Blair JMA, Webber MA, Baylay AJ, Ogbolu DO, Piddock LJV. Molecular mechanisms of antibiotic resistance. Nature Reviews Microbiology. 2015;13(1):42–51. doi: 10.1038/nrmicro3380 25435309

[34] Ledzewicz U, Schättler H. Drug resistance in cancer chemotherapy as an optimal control problem. Discrete and Continuous Dynamical Systems—Series B. 2006;6(1):129–150. doi: 10.3934/dcdsb.2006.6.129

[35] Hansen E, Karslake J, Woods RJ, Read AF, Wood KB. Antibiotics can be used to contain drug-resistant bacteria by maintaining sufficiently large sensitive populations. PLoS Biology. 2020;18(5):1–20. doi: 10.1371/journal.pbio.3000713 32413038 PMC7266357

[36] Lenhart S, Workman JT. Optimal Control Applied to Biological Models; 2007.

[37] Liu LL, Li F, Pao W, Michor F. Dose-dependent mutation rates determine optimum erlotinib dosing strategies for EGFR mutant non-small cell lung cancer patients. PLoS ONE. 2015;10(11):1–17. doi: 10.1371/journal.pone.0141665 26536620 PMC4633116

[38] Greene JM, Gevertz JL, Sontag ED. Mathematical Approach to Differentiate Spontaneous and Induced Evolution to Drug Resistance During Cancer Treatment. JCO Clinical Cancer Informatics. 2019;(3):1–20. doi: 10.1200/CCI.18.00087 30969799 PMC6873992

[39] Greene JM, Sanchez-Tapia C, Sontag ED. Mathematical details on a cancer resistance model. Frontiers in Bioengineering and Biotechnology. 2020;8. doi: 10.3389/fbioe.2020.00501 32656186 PMC7325889

[40] Alexander HK, MacLean RC. Stochastic bacterial population dynamics restrict the establishment of antibiotic resistance from single cells. Proceedings of the National Academy of Sciences of the United States of America. 2020;117(32):19455–19464. doi: 10.1073/pnas.1919672117 32703812 PMC7431077

[41] Xue B, Leibler S. Bet Hedging against Demographic Fluctuations. Physical Review Letters. 2017;119(10):1–5. doi: 10.1103/PhysRevLett.119.108103 28949168

[42] Yoshida M, Reyes SG, Tsuda S, Horinouchi T, Furusawa C, Cronin L. Time-programmable drug dosing allows the manipulation, suppression and reversal of antibiotic drug resistance in vitro. Nature Communications. 2017;8. doi: 10.1038/ncomms15589 28593940 PMC5472167

[43] Newton PK, Ma Y. Nonlinear adaptive control of competitive release and chemotherapeutic resistance. Physical Review E. 2019;99(2):1–10. doi: 10.1103/PhysRevE.99.022404 30934318 PMC7515604

[44] Fischer A, Vázquez-García I, Mustonen V. The value of monitoring to control evolving populations. Proceedings of the National Academy of Sciences of the United States of America. 2015;112(4):1007–1012. doi: 10.1073/pnas.1409403112 25587136 PMC4313848

[45] Vasse M, Bonhoeffer S, Frenoy A. Ecological effects of stress drive bacterial evolvability under sub-inhibitory antibiotic treatments. bioRxiv. 2020; 10.1101/2020.06.30.181099. PMC9723650 37938266

[46] Enriquez-Navas PM, Kam Y, Das T, Hassan S, Silva A, Foroutan P, et al. Exploiting evolutionary principles to prolong tumor control in preclinical models of breast cancer. Science Translational Medicine. 2016;8(327). doi: 10.1126/scitranslmed.aad7842 26912903 PMC4962860

[47] Zhang J, Cunningham JJ, Brown JS, Gatenby RA. Integrating evolutionary dynamics into treatment of metastatic castrate-resistant prostate cancer. Nature Communications. 2017;8(1):1–9. doi: 10.1038/s41467-017-01968-5 29180633 PMC5703947

[48] Hansen E, Read AF. Cancer therapy: Attempt cure or manage drug resistance? Evolutionary Applications. 2020;13(7):1660–1672. doi: 10.1111/eva.12994 32821276 PMC7428817

[49] Shaw LP, Bassam H, Barnes CP, Walker AS, Klein N, Balloux F. Modelling microbiome recovery after antibiotics using a stability landscape framework. ISME Journal. 2019;13(7):1845–1856. doi: 10.1038/s41396-019-0392-1 30877283 PMC6591120

[50] Sharma D, Misba L, Khan AU. Antibiotics versus biofilm: An emerging battleground in microbial communities. Antimicrobial Resistance and Infection Control. 2019;8(1):1–10. doi: 10.1186/s13756-019-0533-3 31131107 PMC6524306

[51] Mansky LM, Pearl DK, Gajary LC. Combination of Drugs and Drug-Resistant Reverse Transcriptase Results in a Multiplicative Increase of Human Immunodeficiency Virus Type 1 Mutant Frequencies. Journal of Virology. 2002;76(18):9253–9259. doi: 10.1128/JVI.76.18.9253-9259.2002 12186909 PMC136424

[52] Frenoy A, Bonhoeffer S. Death and population dynamics affect mutation rate estimates and evolvability under stress in bacteria. PLoS Biology. 2018;16(5):1–23. doi: 10.1371/journal.pbio.2005056 PMC5966242 29750784

[53] Abascal F, Harvey LMR, Mitchell E, Lawson ARJ, Lensing SV, Ellis P, et al. Somatic mutation landscapes at single-molecule resolution. Nature. 2021;593(November 2020). 33911282 10.1038/s41586-021-03477-4

[54] Anttila JV, Shubin M, Cairns J, Borse F, Guo Q, Mononen T, et al. Contrasting the impact of cytotoxic and cytostatic drug therapies on tumour progression. PLoS Computational Biology. 2019;15(11):1–18. doi: 10.1371/journal.pcbi.1007493 31738747 PMC6886869

[55] Stengel RF. Stochastic optimal control: theory and application. John Wiley and Sons, New York; 1986.

[56] Rossetti V, Filippini M, Svercel M, Barbour AD, Bagheri HC. Emergent multicellular life cycles in filamentous bacteria owing to density-dependent population dynamics. Journal of the Royal Society Interface. 2011. doi: 10.1098/rsif.2011.0102 21593029 PMC3203479

