# Supplementary figures and images for: Drug-induced resistance evolution necessitates less aggressive treatment
Source: PLoS Comput Biol. 2021 Sep 23;17(9):e1009418. doi: 10.1371/journal.pcbi.1009418 (PMC8491903; doi:10.1371/journal.pcbi.1009418)

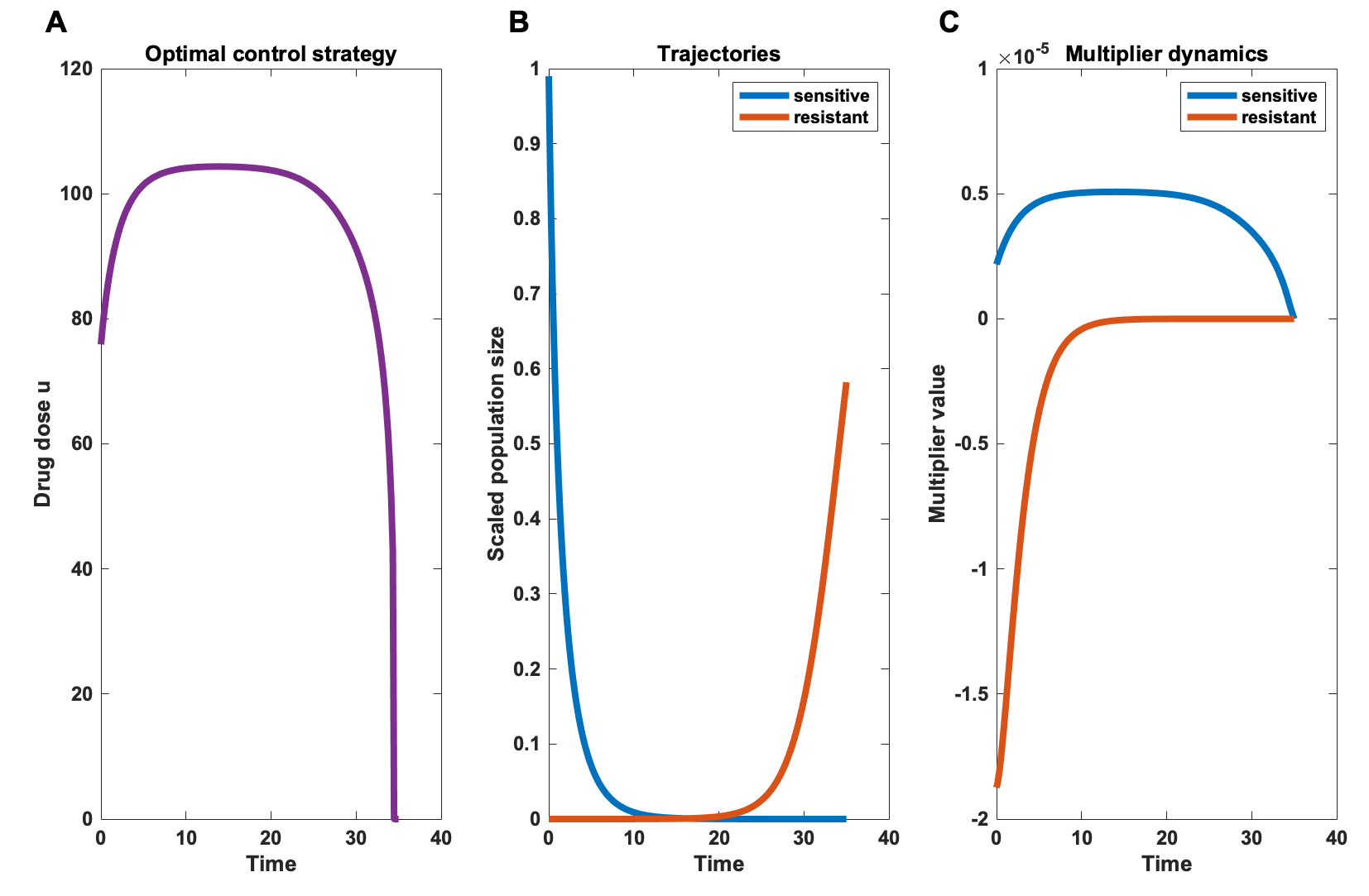

Supplement: S1 Fig — Solution of the optimal control problem (3) using Forward-Backward Sweep Method with parameter values specified in Table 1 (main text). A Optimal treatment strategy uopt(t) as a function of time. B The optimally controlled trajectories S(t) and R(t). In deterministic dynamics the population always experiences an evolutionary rescue. C The dynamics of the multipliers λS(t) and λR(t) corresponding to the sensitive and resistant cells respectively. The multiplier values can be interpreted as sensitivities of the optimal cost C(uopt) to the perturbations in the respective state variables. (TIF) [file pcbi.1009418.s001.tif]

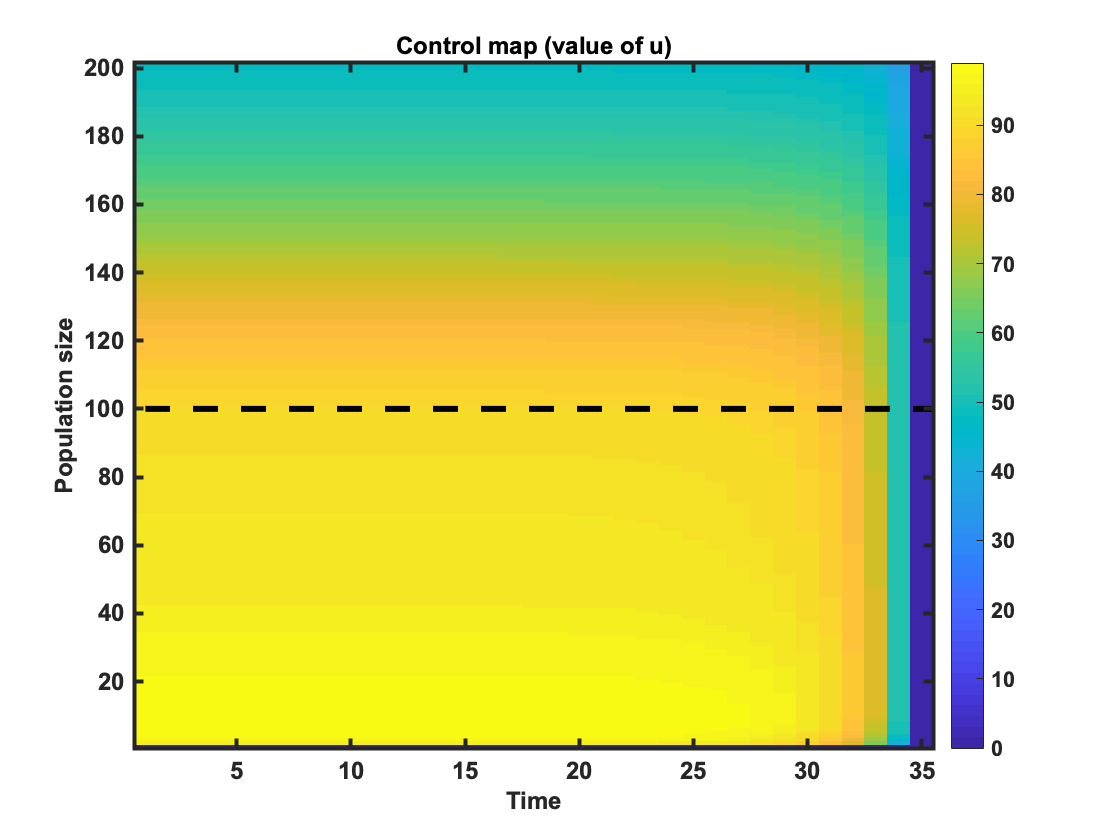

Supplement: S2 Fig — Here the color denotes the optimal control to be applied at the given population size and time. The carrying capacity has been scaled to K = 100. Notice, how the control values start to change in time only at the end of control period, when t > 20. (TIF) [file pcbi.1009418.s002.tif]

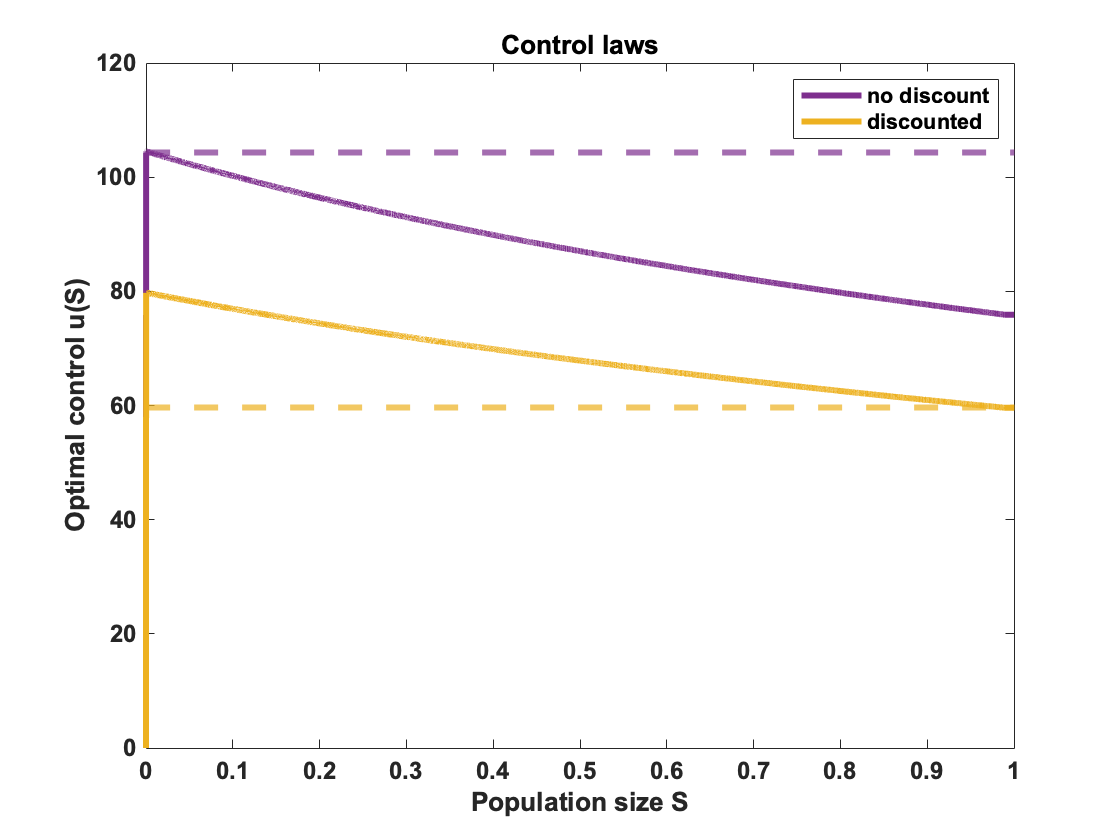

Supplement: S3 Fig — Feedback controls u(S) for the two optimal control problems obtained via the inverse function method. The dashed lines give the optimal constant doses, respectively. The analytically derived stationary profile matches the numerical solution, but cannot be applied to the discounted problem due to the explicit time-dependence. (TIF) [file pcbi.1009418.s003.tif]
