# Supplementary material for: Drug-induced resistance evolution necessitates less aggressive treatment
Source: PLoS Comput Biol. 2021 Sep 23;17(9):e1009418. doi: 10.1371/journal.pcbi.1009418 (PMC8491903; doi:10.1371/journal.pcbi.1009418)
Supplement: S1 Text — (PDF) [file pcbi.1009418.s004.pdf]

S1 Text

Drug-induced resistance evolution necessitates less aggressive treatment

Teemu Kuosmanen, Johannes Cairns, Robert Noble, Niko Beerenwinkel, Tommi Mononen, Ville Mustonen

Solving the optimal controls using Pontryagin's minimum principle

Consider the optimal control problem

$$\min_{u\in\mathcal{U}} \int_0^T S(t,u(t))\mu(u)dt, \quad \mathcal{U}=[0,u_{\mathrm{MTD}}] \tag{1}$$

with $\mu(u)=\mu_0+\alpha u(t)$, subject to the dynamics

$$\begin{cases} \dot{S}=f=r_S(1-\frac{S+R}{K})S-d(u)S-\mu(u)S; \quad S(0)=1 \\ \dot{R}=g=r_R(1-\frac{S+R}{K})R+\mu(u)S; \quad R(0)=0. \end{cases} \tag{2}$$

where $d(u)=d_{\max}\big(1-\frac{1}{1+(\frac{u}{h})^k}\big)$ is fixing the pharmacodynamics of the drug. The problem can be solved with two alternative methods based either on Pontryagin's minimum principle or Hamilton-Jacobi-Bellman equation. The Pontryagin's minimum principle is based on the variational approach and proceeds by defining the Hamiltonian as

$$H=L(t,S,u)+\lambda_1 f+\lambda_2 g, \tag{3}$$

where $L(t,S,u):=S(t,u(t))\mu(u)$ is the Lagrangian cost functional and the multipliers (costate variables) satisfy the following equations:

$$\begin{cases} \dot{\lambda_1} &= -H_S = -\mu(u)-\lambda_1\Big(r_S(1-\frac{2S+R}{K})-d(u)-\mu(u)\Big)-\lambda_2\Big(\mu(u)-r_R\frac{R}{K}\Big); \\ \dot{\lambda_2} &= -H_R = \lambda_1 r_S\frac{S}{K}-\lambda_2\Big(r_R(1-\frac{2R+S}{K})\Big), \end{cases} \tag{4}$$

with boundary conditions

$$\lambda_1(T)=0=\lambda_2(T). \tag{5}$$

The optimal control strategy $u_{\mathrm{opt}}(t)$ is found by studying the function

$$H_u(t)=S(t)\Big(\alpha-\lambda_1\big(d'(u)+\alpha\big)+\lambda_2\alpha\Big), \tag{6}$$

whose roots, should they exist, determine the singular controls. (If no roots exists, then the optimal control reduces to so-called *bang-bang* controls.) Using the parameters given in Table 1 (in main text), two separate roots appear; the first root can be excluded using the Legendre-Clebsch condition ($H_{uu}<0$) [1]. The candidate optimal control $u^*(S,R,\lambda_1,\lambda_2)$ must then be iterated using e.g. the Forward-Backward-Sweep Method [2], where the state variables are first solved *forward* in time using dynamics (2) and then the multipliers are solved *backwards* in time using dynamics (4) with the updated variables. The numerical solution to the problem together with the trajectories and multipliers are showed in S1 Fig.

Time-independent control laws from Hamilton-Jacobi-Bellman equation

Notice that since the Lagrangian cost functional $L(t, S, u)$ does not depend on the number of resistant cells R, we need not explicitly track them. This is because we assume resistant cells are initially so rare that the competitive interactions on the sensitive cell growth is negligible (note that the opposite is not true). If this assumption would be violated, then the cost functional itself would lose its usefulness and the object of the treatment should rather focus in the containment and management of the already present resistance and not its *de novo* emergence. Consequently, we can concentrate our analysis on the single state variable S and assume that its dynamics is independent of R.

To gain further insight, we then solved the same problem using the alternative method based on Hamilton-Jacobi-Bellman (HJB) equation. This approach relies on solving the *cost-to-go* function $J(t, S)$ from the partial differential equation:

$$-\partial_t J(t,S) = \min_{u\in\mathcal{U}} \Big(L(t,S,u) + f(t,S,u)\partial_S J(t,S) \Big) \tag{7}$$

with boundary condition

$$J(T,S) = 0. \tag{8}$$

By recording the minimizing control at each point, the HJB approach gives a *control map*

$$u^*(t,S) = \arg\min_{u\in\mathcal{U}} \Big(L(t,S,u) + f(t,S,u)\partial_S J(t,S) \Big), \tag{9}$$

from which the optimal control can be read for any conceivable state. The HJB equation above is for the deterministic version of the control problem that corresponds to the Pontryagin methods described above. We also solved the stochastic version of the problem, and found that demographic stochasticity plays very little role here. The resulting control map is showed in S2 Fig.

We notice from S2 Fig. that the optimal control is virtually independent of time, except at the very end of the control period, when the control is discontinued. Closer inspection of this reveals that this is in fact a biologically irrelevant boundary effect created by the fixed end-time corresponding to stopping therapy and letting the target population grow again. Consequently, by letting the end-time T to be sufficiently large so that the sensitive population is almost surely eliminated before the end, then the optimal treatment becomes time-independent and we can solve for a closed-loop control $u(S)$ which has only feedback from the current population size. Such stationary solution can be obtained analytically for the deterministic HJB by setting

$$-\partial_t J(t,S) = \min_{u\in\mathcal{U}} \Big(L(t,S,u) + f(t,S,u)\partial_S J(t,S) \Big) = 0 \tag{10}$$

and carrying out the minimisation by formally differentiating the terms inside the brackets with respect to u. This minimization yields condition

$$\partial_S J(t,S) = -\frac{L_u(t,S,u)}{f_u(t,S,u)}, \tag{11}$$

which can be substituted back to the HJB equation. The *stationary profile* for the problem then reads

$$L(t,S,u) - f(t,S,u)\frac{L_u(t,S,u)}{f_u(t,S,u)} = 0, \tag{12}$$

which gives an *implicit* equation for the optimal control. Furthermore, as the time-dependence realizes only via the state and control variables, solving the implicit

equation yields a time-independent control law $u(S)$. Indeed, by substituting the Lagrangian cost functional and the sensitive dynamics, we get

$$S\mu(u) - S\big(r(S) - d(u) - \mu(u)\big)\frac{S\mu'(u)}{-S\big(d'(u) + \mu(u)\big)} = 0$$
$$\mu(u) + \big(r(S) - d(u) - \mu(u)\big)\frac{\mu'(u)}{d'(u) + \mu'(u)} = 0.$$

Notice that here we assumed nothing about the precise functional form of the dose-dependent mutation rate $\mu(u)$, the pharmacodynamics $d(u)$ or the density-dependent growth model $r(S)$ except that these are all differentiable functions with respect to u. Furthermore, notice that as the state variables S cancel out, the only remaining dependence of the state variable happens via the assumed density dependent growth model, where we assumed that $r(N) \approx r(S)$. Thus, we have now derived the control law equation, where the only technical modelling assumption we have made is that of the *log-kill hypothesis*, where the control leverage depends only of the drug concentration and specifically does not depend on the population size.

The analytically derived stationary profile cannot be directly applied to the discounted problem, because of its explicit time-dependence. However, the same stationary profile can be obtained numerically more simply if the optimal time-dependent control eliminates the target population without allowing it to grow in between. Then $S(t)$ is a monotonically decreasing function and hence there exists an inverse function $S^{-1} : [0, 1] \to [0, T]$ which gives the time at which the population was at any given size. Now, if indeed time-independent control law $u(S)$ does exist, it must be unique and thus the optimal control for some population size S' must satisfy $u(S') = u_{\mathrm{opt}}(t')$ where $S(t') = S'$. Then the stationary profile can be obtained using the inverse function as

$$u(S') = u_{\mathrm{opt}}(S^{-1}(S')).$$

Applying this inverse function method agrees with the analytically derived stationary profile and can be used also to the discounted problem (see S3 Fig.).

Here we have focused on the problem of determining the optimal way of eliminating the target population, with respect to two biologically meaningful objectives. The derived optimal treatment strategies were calculated with respect to the simple constraint that there is some maximum tolerated dose u_{MTD}, which may be below the drug concentration where the pharmocodynamical effect is assumed to have plateaued. Even though the cumulative drug concentration was not constrained, we did consider that the constant MTD would generally be lower than $u_{\max}$ (where the death rate saturates) when demonstrating the effect sizes compared to the optimal treatment. Such constraints can also be easily incorporated to the presented optimal control framework by appending the Hamiltonian with an additional state variable, which enforces the isoperimetric constraint (see e.g. [3], [4]).

References

1. Schättler H, Ledzewicz U. Optimal Control for Mathematical Models of Cancer Therapies. vol. 42. Springer; 2015.
2. Lenhart S, Workman JT. Optimal Control Applied to Biological Models; 2007.
3. Hamdache A, Elmouki I, Saadi S. Optimal control with an isoperimetric constraint applied to cancer immunotherapy. International Journal of Computer Applications. 2014;94(15).

4. Stengel RF. Stochastic optimal control: theory and application. John Wiley and Sons, New York; 1986.
